# A modifier screen identifies regulators of cytoskeletal architecture as mediators of Shroom-dependent changes in tissue morphology

**DOI:** 10.1242/bio.055640

**Published:** 2021-02-03

**Authors:** Jeffrey D. Hildebrand, Adam D. Leventry, Omoregie P. Aideyman, John C. Majewski, James A. Haddad, Dawn C. Bisi, Nancy Kaufmann

**Affiliations:** Department of Biological Sciences, University of Pittsburgh, Pittsburgh, PA 15260, USA

**Keywords:** Shroom, Cytoskeleton, *Drosophila*, Morphogenesis, Epithelia

## Abstract

Regulation of cell architecture is critical in the formation of tissues during animal development. The mechanisms that control cell shape must be both dynamic and stable in order to establish and maintain the correct cellular organization. Previous work has identified Shroom family proteins as essential regulators of cell morphology during vertebrate development. Shroom proteins regulate cell architecture by directing the subcellular distribution and activation of Rho-kinase, which results in the localized activation of non-muscle myosin II. Because the Shroom-Rock-myosin II module is conserved in most animal model systems, we have utilized *Drosophila melanogaster* to further investigate the pathways and components that are required for Shroom to define cell shape and tissue architecture. Using a phenotype-based heterozygous F1 genetic screen for modifiers of Shroom activity, we identified several cytoskeletal and signaling protein that may cooperate with Shroom. We show that two of these proteins, Enabled and Short stop, are required for ShroomA-induced changes in tissue morphology and are apically enriched in response to Shroom expression. While the recruitment of Ena is necessary, it is not sufficient to redefine cell morphology. Additionally, this requirement for Ena appears to be context dependent, as a variant of Shroom that is apically localized, binds to Rock, but lacks the Ena binding site, is still capable of inducing changes in tissue architecture. These data point to important cellular pathways that may regulate contractility or facilitate Shroom-mediated changes in cell and tissue morphology.

## Abstract (precis)

**Summary:** Using *Drosophila* as a model system, we identify F-actin and microtubules as important determinants of how cells and tissues respond to Shroom induced contractility.

## INTRODUCTION

Tissue architecture is typically defined during specific stages of embryonic development and errors in these processes can result in human disease. One example is formation of the vertebrate neural tube. The neural tube is formed via the concerted effort of many cellular pathways that functionally convert a plate of neural ectoderm into a closed tube. Errors in this process can result in birth defects such as spina bifida, exencephaly, or craniorachischisis (Nikolopoulou et al., 2017). One cellular pathway that controls this process is regulated by the Shroom3 cytoskeletal adaptor protein (Haigo et al., 2003; Hildebrand and Soriano, 1999). Shroom3 controls neural tube morphogenesis via the formation of apically positioned contractile networks of actomyosin and these networks facilitate neural tube closure by inducing apical constriction and the anisotropic contraction of actin filaments (Haigo et al., 2003; Hildebrand, 2005; McGreevy et al., 2015). This is accomplished via the modular nature of Shroom3. Shroom3 localizes to the apical compartment of epithelial adherens junctions via a direct interaction with F-actin (Hildebrand and Soriano, 1999). This interaction is mediated by the Shroom Domain (SD) 1, a unique actin-binding motif present in most Shroom proteins characterized to date (Dietz et al., 2006; Hildebrand and Soriano, 1999; Yoder and Hildebrand, 2007). Shroom3 function is also dependent on Rho-kinase (Rock), such that Shroom3 directly binds to Rock and regulates both its localization and catalytic activity (Das et al., 2014; Nishimura and Takeichi, 2008; Zalewski et al., 2016). The interaction between Shroom and Rock has been elucidated at the molecular level and is mediated by the conserved SD2 region of Shroom and a conserved coiled-coil region of Rock (Mohan et al., 2013, 2012; Zalewski et al., 2016). The interaction between Shroom and Rock results in the localized activation of non-muscle myosin II (myosin II) contractility, which provides the mechanical force needed to facilitate neural tube morphogenesis. The regulation of myosin II activity by Rock and other cellular pathways has been well described (Heissler and Sellers, 2016). Rock modulates myosin II activity in two ways. First, Rock can directly phosphorylate the associated regulatory light chain (RLC), which modulates the actin-associated ATPase activity and the conformation of myosin II (Amano et al., 1996). Secondly, Rock negatively regulates the phosphatase that dephosphorylates the RLC, thus preventing the inactivation of myosin II (Feng et al., 1999).

Shroom proteins are required for numerous biological processes and are associated with several human diseases. In mammals, there are three definitive Shroom proteins, Shroom2, Shroom3, and Shroom4, each of which contains an N-terminal PDZ domain, the centrally located SD1, and the C-terminally located SD2 (Dietz et al., 2006; Hildebrand and Soriano, 1999; Yoder and Hildebrand, 2007). All three proteins can directly interact with F-actin and regulate cell morphology via Rock (Dietz et al., 2006; Farber et al., 2011; Hildebrand, 2005; Yoder and Hildebrand, 2007). In humans, *SHROOM2* has been linked to neural tube morphogenesis, colorectal cancer, and medulloblastoma (Chen et al., 2018; Dunlop et al., 2012; Shou et al., 2015), while *in vitro* studies indicate it is important for cell migration, vasculogenesis, metastasis, and melanosome biogenesis (Fairbank et al., 2006; Farber et al., 2011; Yuan et al., 2019). *SHROOM3* mutations have been implicated in chronic kidney disease, heart morphogenesis, and neural tube closure in humans (Deshwar et al., 2020; Durbin et al., 2020; Köttgen et al., 2009; Lemay et al., 2015; Matsuura et al., 2020; Tariq et al., 2011). Using model organisms or cell culture, Shroom3 has been shown to control neural tube closure, axon growth, intestine architecture, eye morphogenesis, thyroid budding, and kidney development (Grosse et al., 2011; Hildebrand and Soriano, 1999; Khalili et al., 2016; Loebel et al., 2016; Plageman et al., 2010; Taylor et al., 2008; Yeo et al., 2015). Finally, *SHROOM4* mutations have been associated with X-linked mental defects (Armanet et al., 2015; Danyel et al., 2019; Zapata et al., 2017).

We have shown that the *Shroom* gene is conserved in *Drosophila* and encodes multiple protein isoforms that have different subcellular distributions and activities *in vivo* (Bolinger et al., 2010). The most highly conserved region of *Drosophila* Shroom is the SD2, the region that binds to *Drosophila* Rho-kinase (Rok) (Mohan et al., 2012). *Drosophila* Shroom also contains a divergent SD1 motif and this appears to mediate localization to adherens junctions in polarized epithelia (Bolinger et al., 2010). Consistent with the known activities of mammalian Shroom3, expression of *Drosophila* Shroom in epithelial cells induces apical constriction in a Rok and myosin II dependent manner (Bolinger et al., 2010). While Shroom3 is essential for mouse and human development, Shroom is not absolutely essential for *Drosophila* viability, as *Shroom* null flies can be recovered, albeit with significantly reduced frequency (Simoes Sde et al., 2014). In *Drosophila* embryos, Shroom is planarly distributed and works in a complicated network with RhoA, Rok, and myosin II to control convergent extension movements (Simoes Sde et al., 2014). These elegant studies showing the role of Shroom in regulating directional contractility are supported by observations that Shroom proteins can be polarly distributed in mammalian tissues and cells (Farber et al., 2011; McGreevy et al., 2015; Muccioli et al., 2016).

To better understand the mechanisms that control Shroom-regulated changes in cell and tissue morphology, we have established tools to perform genetic screens for modifiers of Shroom activity in *Drosophila*. Shroom gain-of-function phenotypes in the eye and wing can be suppressed or enhanced by known components of the Shroom pathway. Using a candidate approach, we have identified several cytoskeletal regulators, including Short stop and Enabled, as participants in Shroom-mediated changes in cell morphology. Shroom regulates the distribution of Ena and this is likely mediated by conserved proline-rich sequences in Shroom and the EVH1 domain of Ena. We further show that while Ena is required for the Shroom gain-of-function phenotypes, apical recruitment of Ena is not sufficient to cause changes in cell morphology. Additionally, by using an isoform of Shroom that does not bind Ena, but still engages Rok, we show that apical constriction can be modulated by different cellular pathways depending on the context.

## RESULTS

### Expression of Shroom disrupts normal eye and wing development

**Fig. 1.**
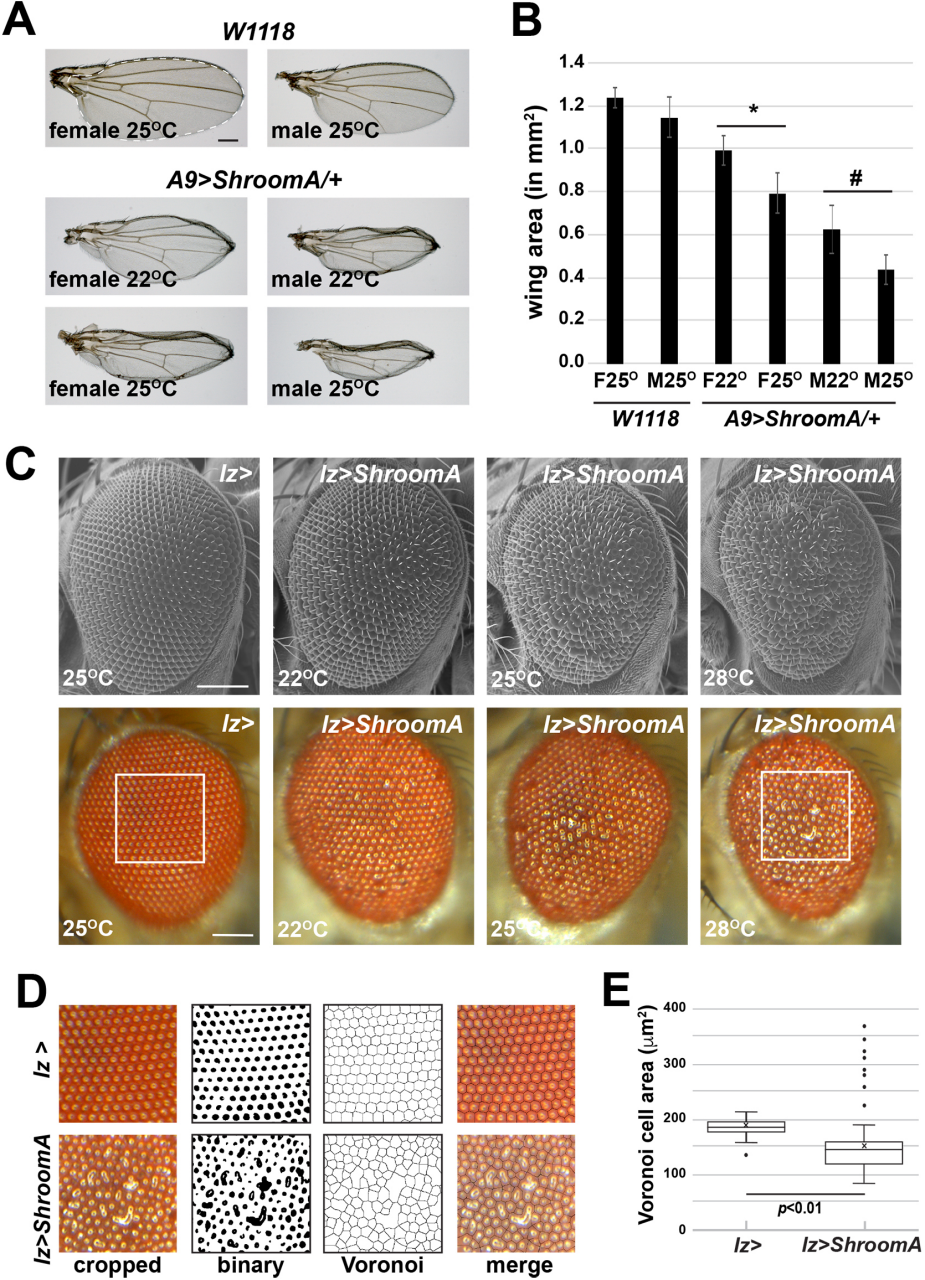
**ShroomA expression perturbs normal tissue morphology.** (A) Wings from control (W1118) or F1 heterozygous adults from crosses of homozygous *A9>ShroomA* females to male *W1118* flies performed at either 22°C or 25°C. Scale bar: 100 µm. (B) Quantification of wing blade area (white dotted line in A). *n*=at least 15 wings, error bars show standard deviation (s.d.), * and # denote *P*<0.01 relative to female (F) and male (M) *W1118* controls, respectively. (C) Eyes from control (*lz>*) or *lz>ShroomA/+* male flies raised at the indicated temperatures and imaged with either electron or light microscopy. Scale bars: 100 µm. (D) Steps used to quantify the rough eye phenotype. Cropped images (boxed regions in C) were converted to binary and processed to Voronoi tessellation using the ommatidia reflections as Voronoi generators. Merged images show the overlay of the tessellation and the cropped image. Areas of the generated Voronoi cells were measured and box and whisker plots used to display the distribution of cell areas, *n*=5 eyes and at least 75 Voronoi cells per eye. Significance was determined using a two-tailed *t*-test.

**Fig. 2.**
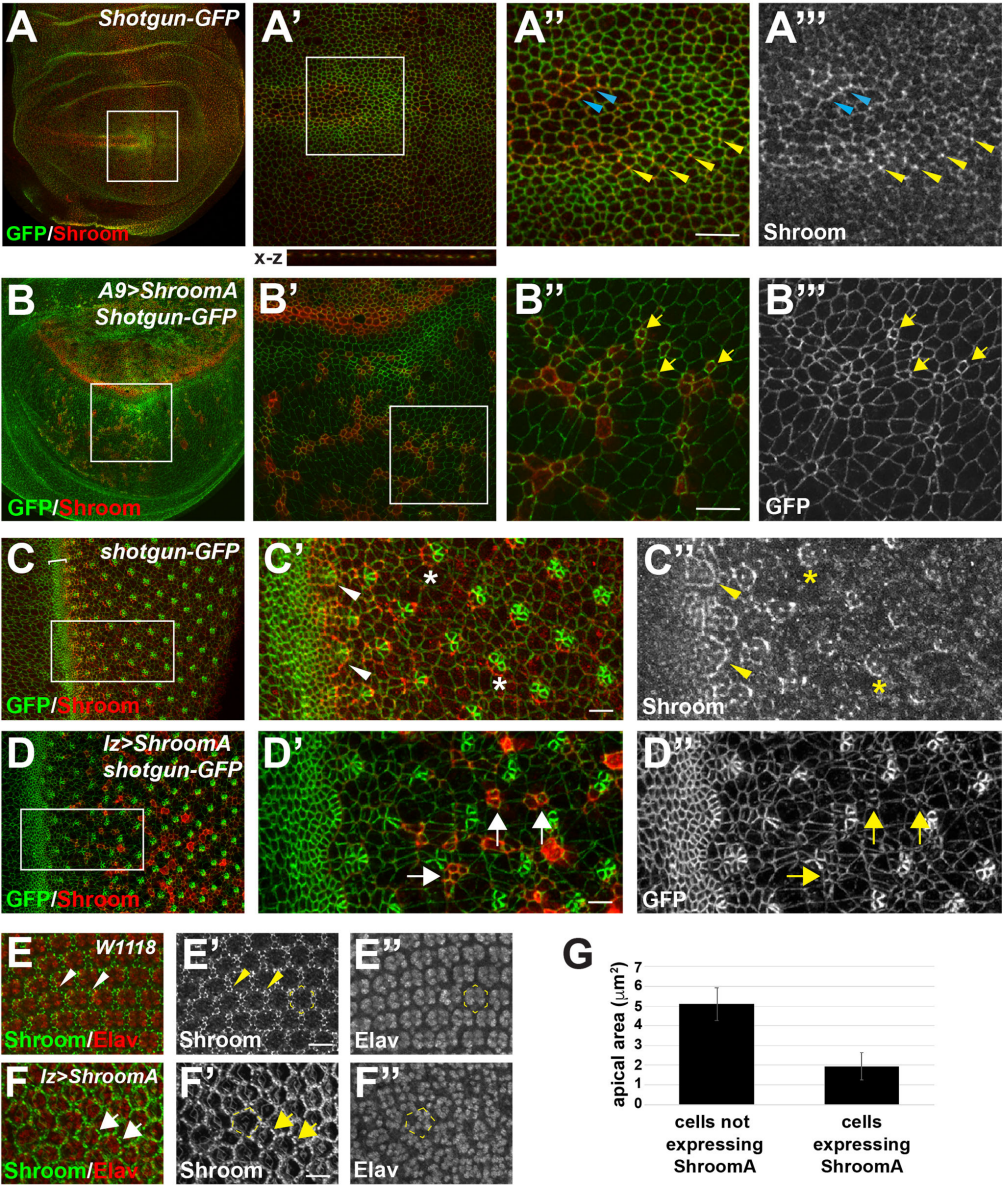
**Ectopic ShroomA expression alters cell and tissue architecture.** (A,B) Wing imaginal discs from *Shotgun-GFP/+* (A) or *A9>ShroomA/+;Shotgun-GFP/+* (B) larva were isolated and stained to detect Shroom and GFP. Dorsal to the top, anterior to the left, boxed regions are shown enlarged in A′–A‴. Endogenous ShroomA is localized with E-cadherin in apically localized adherens junctions (A′, X-Z projection) and in cell–cell and tricellular junctions (A″ and A‴, blue and yellow arrowheads, respectively). Ectopic ShroomA is highly expressed in the dorsal compartment of the wing pouch (B). Cells expressing excess ShroomA are apically constricted (arrows in B″ and B‴). (C,D) Eye imaginal discs from *Shotgun-GFP/+* (C–C″) or *lz>ShroomA/+;Shotgun-GFP/+* (D–D″) larva were isolated and stained to detect Shroom and GFP, anterior is to the left. Endogenous Shroom protein is detected in the posterior portion of the morphogenic furrow (C, bracket), the forming ommatidia, and becomes restricted to R3/4 (C′ and C″, arrowheads and asterisk, respectively). Ectopic ShroomA is expressed posterior to the morphogenetic furrow in the interommatidial cells. Cells expressing exogenous ShroomA display apical constriction and unequal distribution of GFP in the cell junctions (D′ and D″, arrows). (E,F) Retinas from control *W1118* (E) or *lz>ShroomA/+* (F) pupae were isolated and stained to detect Shroom and Elav. In controls, endogenous Shroom protein (E′) is expressed in the pigment cells and is localized to a subset of cell–cell junction and tricellular junctions (arrowheads). Elav staining shows the organization of the photoreceptors within the hexagonal ommatidia based on pigment cell positioning (E″, dotted outline). In *lz>ShroomA/+* retinas (F), exogenous ShroomA is expressed in the pigments cells and these cells appear to be disorganized and apically constricted (F′, arrows). Photoreceptor organization is also perturbed based on Elav staining relative to positioning of the pigment cells (F″, dotted outline). (G) Measurements of the apical areas of wing imaginal disc cells that either do or do not over express ShroomA, based on panels B″ and B‴. Significance was determined using a two-tailed *t*-test, error bars show the s.d. Scale bars: 10 µm in all panels.

We hypothesized that imaginal discs from *Drosophila melanogaster* are a relevant model for assessing Shroom function during cell and tissue morphology since they are comprised of polarized epithelia and endogenously express the ShroomA protein (Fig. 2), the isoform most similar to mammalian Shroom3 (Bolinger et al., 2010). To perform these studies, we generated lines expressing ShroomA in the wing or eye imaginal discs under control of *A9-gal* (*A9*) or *lozenge-gal4* (*lz*), respectively. Over expression of ShroomA in the eye or wing imaginal discs results in rough eye and crumpled wing phenotypes in the adult flies. Wings from *A9>ShroomA/+* flies are significantly smaller and show dorsal curling at the margin (Fig. 1A). There do not appear to be defects in the differentiation of the various cell types of the wing, as the bristles and veins exhibit normal morphology and positioning. Wing phenotypes were quantified by measuring the total area of the wing blade that is derived from the wing pouch of the imaginal disc (Fig. 1B).

Adult male *lz>ShroomA/+* flies exhibit a rough eye phenotype and the eye is typically smaller (Fig. 1C). To quantify the severity of the eye phenotype, images were cropped to include the center portion of the eye and converted to a binary image representing the reflections of each ommatidia (Fig. 1D). The binary image was processed to a Voronoi tessellation using the reflections of the ommatidia as the generators. All steps were performed using ImageJ. This and similar approaches have been used to determine the distribution and geometry of tissue structures and cells both *in vitro* and *in vivo* (Barriga et al., 2013; Cheng et al., 2014; Matsushima et al., 2015). The Voronoi cells generated by this conversion are representative of the distribution of ommatidia in the original sample, as they capture features such as fused and misaligned ommatidia (Fig. 1D, merge). The area of the Voronoi cells was measured and these values used as a quantitative measure of the rough eye phenotype (Fig. 1E).

Previous studies suggest the Gal4-UAS expression system is more effective at higher temperatures (Brand and Perrimon, 1993; Carvajal-Gonzalez et al., 2016). Additionally, because the transgenes used for these studies are located on the X-chromosome, we hypothesized there could be phenotypic variation in males versus females due to differences in expression. We therefore assessed the Shroom-induced phenotypes at different temperatures in males and females. Wings from *A9>ShroomA/+* flies exhibit a continuum of phenotypes depending on the sex and temperature, with males raised at higher temperatures exhibiting the most severe phenotype (Fig. 1A,B). We observed a similar gradation of the eye phenotype of *lz>ShroomA/+* male flies raised at different temperatures (Fig. 1C). These observations indicate that the eye and wing phenotypes can be utilized in modifier screens since they can be enhanced or suppressed under different conditions.

### Shroom alters cell and tissue morphology

We have shown that Shroom proteins induce apical constriction when expressed in polarized epithelia in both cell culture and *Drosophila* embryos (Bolinger et al., 2010; Hildebrand, 2005). To determine if the observed phenotypes result from aberrant tissue morphology, we isolated imaginal discs from third instar larva from crosses of homozygous A9>*ShroomA* or *lz>ShroomA* females to homozygous *shotgun-GFP* (dE-cadherin) males and stained the resulting F1 heterozygous larva to detect Shroom and GFP (Fig. 2). In the wing pouch of control *shotgun-GFP/+* imaginal discs, we observe endogenous Shroom protein co-localized with GFP in cell–cell and tri-cellular junctions, with highest expression in rows of cells that border the anterior portion of the wing margin and the anterior-posterior boundary (Fig. 2A–A″′). In A9>*ShroomA/+; shotgun-GFP/+* discs, ectopic ShroomA is primarily expressed in the dorsal portion of the wing pouch, the future hinge region, and more sporadically in the ventral compartment (Fig. 2B–B‴). In the ventral portion of the wing imaginal disc, cells expressing ShroomA have smaller apical area, consistent with Shroom's ability to initiate apical constriction (Fig. 2B‴, arrows, G). Collectively, these data indicate that ShroomA expression alters epithelial cell shape in the imaginal disc, resulting in the observed wing phenotype.

In the control *shotgun-GFP/+* eye imaginal disc, endogenous Shroom is expressed in the morphogenetic furrow and the pre-cluster rosettes of the developing ommatidia (Fig. 2C–C″, bracket and arrowheads, respectively). Shroom expression becomes restricted to R3/4 (Fig. 2C″, asterisk) and is eventually lost from the ommatidia. The expression pattern and subcellular distribution of Shroom is similar to that previously described for myosin II in these cells (Escudero et al., 2007; Robertson et al., 2012), consistent with the role of Shroom in modulating myosin II contractility. In *lz*>*ShroomA/+; shotgun-GFP/+* eye discs, ectopic ShroomA is detected in cells posterior to the morphogenetic furrow and is excluded from the ommatidia (Fig. 2D). As seen in the wing imaginal discs, ShroomA-expressing cells appear apically constricted relative to neighboring cells (Fig. 2D′, arrows). Overall, formation of the ommatidia does not appear drastically altered, although some ommatidia are irregular in shape (Fig. 2D′,D″).

To determine if the observed rough eye phenotype is consistent with defects in cellular organization or differentiation, we stained pupal retinas to detect ShroomA and Elav (Fig. 2E,F). Endogenous Shroom protein is expressed in the pigment cells and these cells are organized in a hexagonal array consistent with ommatidia organization (Fig. 2E′). Within these cells, Shroom is localized to cell–cell and tricellular junctions. Elav is expressed in highly organized clusters of photoreceptor cells (Fig. 2E″). In *lz>ShroomA* retinas, ectopic ShroomA is expressed in the pigment cells, with lower expression observed in the cone cells (Fig. 2F′). In these retinas, the regular hexagonal array of pigment cells is disrupted and the ommatidia exhibit irregular shapes and fusions (Fig. 2F and F, dotted outline). The individual pigments cells also appear apically constricted relative to the controls (Fig. 2F′, arrows). This disorganization is further supported by the erratic positioning of the photoreceptors as evidenced by Elav staining (Fig. 2F″). These data indicate that ectopic ShroomA causes errors in formation of the pupal retina that are consistent with the adult rough eye phenotype but does not appear to alter differentiation during eye development.

### A genetic approach to identify modifiers of Shroom activity

**Fig. 3.**
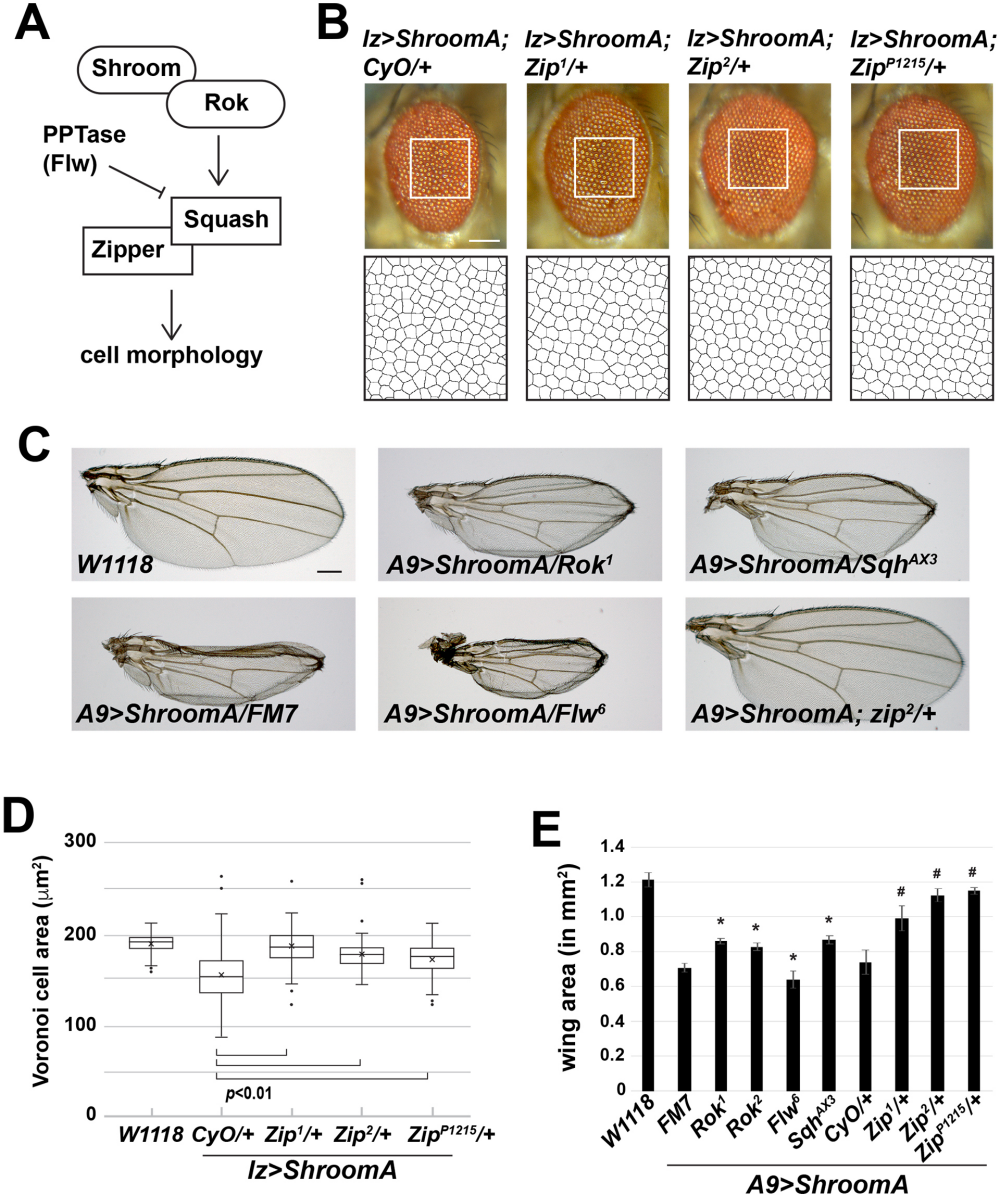
***Zip*, *Sqh*, *Rok*, and *Flw* modify the ShroomA phenotype in adult eyes and wings.** (A) Schematic of the core components of the Shroom pathway. (B) Images of heterozygous F1 male adult eyes of the indicated genotypes from crosses performed at 28°C. Boxed regions are shown enlarged and processed to produce the Voronoi tessellation that was used to quantify the phenotype. (C) Images of heterozygous F1 female adult wings of the indicated genotypes from crosses performed at 25°C. (D) Quantification of the rough eye phenotype. *n*=5 eyes and at least 75 Voronoi cells per eye. (E) Quantification of the wing phenotype, *n*=at least 15 wings, * denotes *P*<0.01 relative to FM7 controls and # denotes *P*<0.01 relative to *CyO* control, error bars show s.d. In both D and E, significance was determined using a two-tailed *t*-test. Scale bars: 100 µm in all panels.

We hypothesized that *A9>ShroomA* and *lz>ShroomA* flies could be utilized to assess the role of other proteins in Shroom-regulated processes. As a proof of principle, we tested if reducing the gene dosage of known components of the Shroom pathway, including *Zipper* (*Zip*, myosin II), *Rho-Kinase* (*Rok*), *Spaghetti Squash* (*Sqh*, RLC), and *Flapwing* (*Flw, PP1β9C* encoding a PP1β type protein also referred to as PP1c) could modify the ShroomA-induced phenotypes described above (Fig. 3A). To accomplish this, we performed a heterozygous F1 modifier screen using mutant alleles of the above genes (see Fig. S1). To assess the role of myosin II in the rough eye phenotype, we crossed homozygous *lz>ShroomA* females to *Zipper/CyO* heterozygous males at 28°C, collected the resultant heterozygous F1 male progeny, and analyzed the rough eye phenotype of *lz>ShroomA*; *CyO/+* (control) versus *lz>ShroomA; Zipper/+* (experimental) flies from the same cross. To eliminate potential complications from background effects, we utilized multiple mutant alleles of *Zipper*. As predicted by our model, reducing the dosage of myosin II protein suppresses the rough eye phenotype caused by ShroomA over expression (Fig. 3B,D).

The location of *Rok*, *Sqh*, and *Flw* on the X-chromosome precluded analysis using *lz>ShroomA* because of the dominant marker *B* associated with the *FM7* balancer. We instead used *A9>ShroomA* to assess the ability of these genes to modify the Shroom phenotype. For X-linked candidates, we crossed females heterozygous for the candidate allele (i.e. *rok^1^/FM7*) to *A9>ShroomA* males and collected trans-heterozygous F1 females of the following genotypic classes: A9>ShroomA/candidate allele (experimental, i.e. *A9>ShroomA/rok^1^*) and A9>ShroomA/balancer (controls, i.e. *A9>ShroomA/FM7*). Consistent with the above results, reducing the dosage of either *zipper*, *rok*, or *sqh* suppresses the phenotype caused by ShroomA overexpression in the wing imaginal disc (Fig. 3C,E). Conversely, reducing the dosage of PP1c (*Flw*) enhances the Shroom phenotype in the wing. One concern was that the dominant *CyO* locus present in most chromosome 2 balancers might interfere with our analysis. However, our data indicate this is not the case as *A9>ShroomA*/*FM7*, *A9>ShroomA*; *CyO/+*, and *A9>ShroomA*/+ females all exhibit equivalent wing phenotypes (Figs 1 and 3). It is interesting to note that while all three alleles of *Zipper* acted as suppressors, they did not produce identical results, with the *Zip^1^* allele being the weakest. Both *Zip1* and *Zip2* are caused by truncation mutations and reported to be loss-of-function alleles (Franke et al., 2010). This suggests that genetic background may influence phenotype modification and that testing multiple alleles will likely be an important step in the screening process. In total, these data indicate that the *A9>ShroomA* and *lz>ShroomA* phenotypes can be modified by second site enhancers and suppressors.

### Candidate-based screen for *Shroom* modifiers

**Fig. 4.**
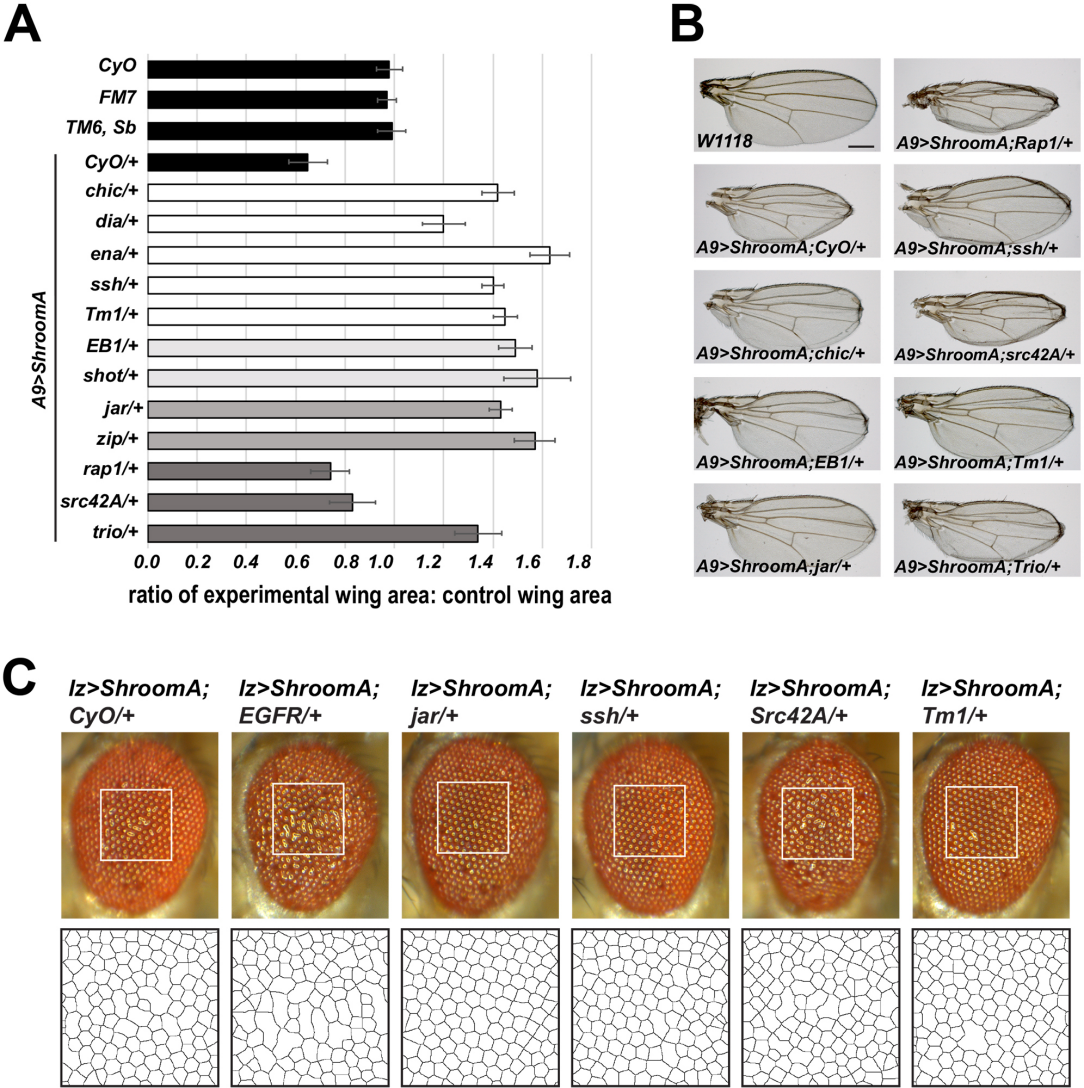
**Results of the heterozygous F1 modifier screen to identify regulators of ShroomA-induced phenotypes.** (A) Graph showing the ratio of the wing areas of F1 experimental (*A9>ShroomA*; mutation/+) to control (*A9>ShroomA*; balancer/+) progeny. Ratios are greater than 1 if the experimental wing is larger than the control (suppressor) and less than 1 if the experimental wing is smaller than the control (enhancer). Black bars represent the ratios of balancer lines to *W1118* wings or *A9>ShroomA/+ to W1118*. Shown are only those crosses that exhibit ratios that are a minimum of 1.5 standard deviations above or below 1. Error bars indicate the s.d. of the ratios. Different shaded bars represent classification by molecular function (actin dynamics, microtube organization, motor function, or signaling). *n*=at least 15 wings per genotype. (B) Representative wings of the indicated genotypes showing enhanced or suppressed phenotypes. (C) Representative enhanced or suppressed eye phenotypes of the indicated genotypes. Boxed regions were used to generate Voronoi tessellations (bottom panels).

To identify other proteins involved in Shroom-mediate changes in cell shape, we performed a heterozygous F1 modifier screen of candidate genes known to encode regulators of cytoskeletal architecture and cell morphology. Crosses were performed as described above (see also Fig. S1). Wings were isolated from F1 heterozygous control and experimental females and measured to define wing areas. To determine if a given candidate enhanced or suppressed the wing phenotype, we calculated the ratio of the areas of experimental (i.e. *A9>ShroomA; Zipper/+*) to control (i.e. *A9>ShroomA*; *CyO/+*) wings. A ratio of 1 indicates there is no difference between the experimental and control groups, a value less than 1 indicates the wing area of the experimental group is smaller than control (enhanced wing phenotype), and a value greater than 1 indicates the wing area of the experimental group is larger than controls (suppressed wing phenotype). We identified 12 genes (in addition to those tested above) as potential modifiers of Shroom function from a pool of 67 unique candidates, with most acting similarly in both the wing and eye disc (Fig. 4; Table S1). While this frequency seems high, we anticipate this is the result of testing candidate genes and this frequency would be much lower on a genome-wide scale. The majority of identified candidates act as suppressors and are predicted to function as regulators of cytoskeletal organization. It is interesting to note that mediators of processes such as cell adhesion or apical-basal polarity did not modify the Shroom-induced phenotypes using this approach. We identified multiple enhancers of Shroom, two tyrosine kinases, EGFR and Src42A, and Rap1, although EGFR was found to modify only the rough eye phenotype. The parental *Rap1^1^* allele used in these studies causes a rough eye phenotype and could not be accurately evaluated in combination with *lz>ShroomA*. EGFR signaling has been shown to act in several aspects of eye development, including both cell fate specification and cell morphology (Brown et al., 2006; Freeman, 1996; Kumar, 2012) while Src42A has been implicated in epithelial morphogenesis via the regulation of adherens junction dynamics (Shindo et al., 2008; Takahashi et al., 2005). Previous work suggests that Rap1 works in cooperation with several factors to define epithelial morphology in *Drosophila* and is required for Shroom3 activity in *Xenopus* neural tube closure (Bonello et al., 2018; Haigo et al., 2003; O'Keefe et al., 2012).

### *Enabled* and *short stop* are required for Shroom-induced phenotypes

**Fig. 5.**
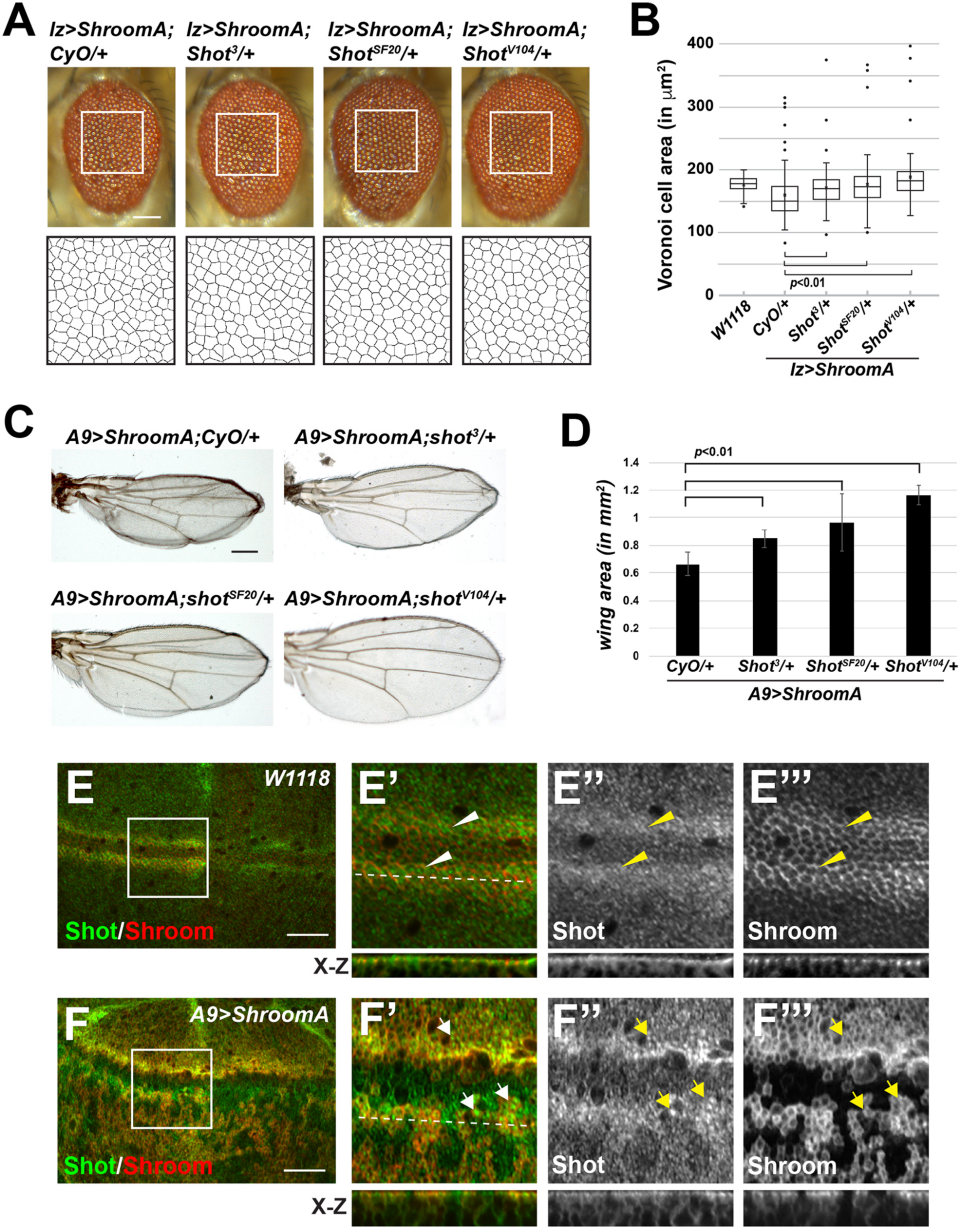
**Short stop participates in ShroomA induced phenotypes.** (A,B) Images and quantification of *lz>ShroomA;shot/+* F1 male adult eyes heterozygous for the indicated alleles crossed at 28°C. Boxed regions were enlarged and used to generate the Voronoi tessellation that was used to quantify the phenotype. (C) Images of heterozygous F1 female adult wings of the indicated genotypes crossed at 25°C. (D) Quantification of wing size for the indicated genotypes. *n*=at least 15 wings. Scale bar: 100 µm. Significance was determined using a two-tailed *t*-test, error bars indicate s.d. (E,F) Wing imaginal discs from control (E, W1118) or *A9>ShroomA* (F) larva stained to detect ShroomA and Shot. Boxed regions in E and F are shown enlarged in subsequent panels. Dotted line, region used to generate X-Z projections; arrowheads, complementary distribution of endogenous Shroom and Shot; arrows, Shot localization in cells overexpressing ShroomA; scale bars: 50 µm.

**Fig. 6.**
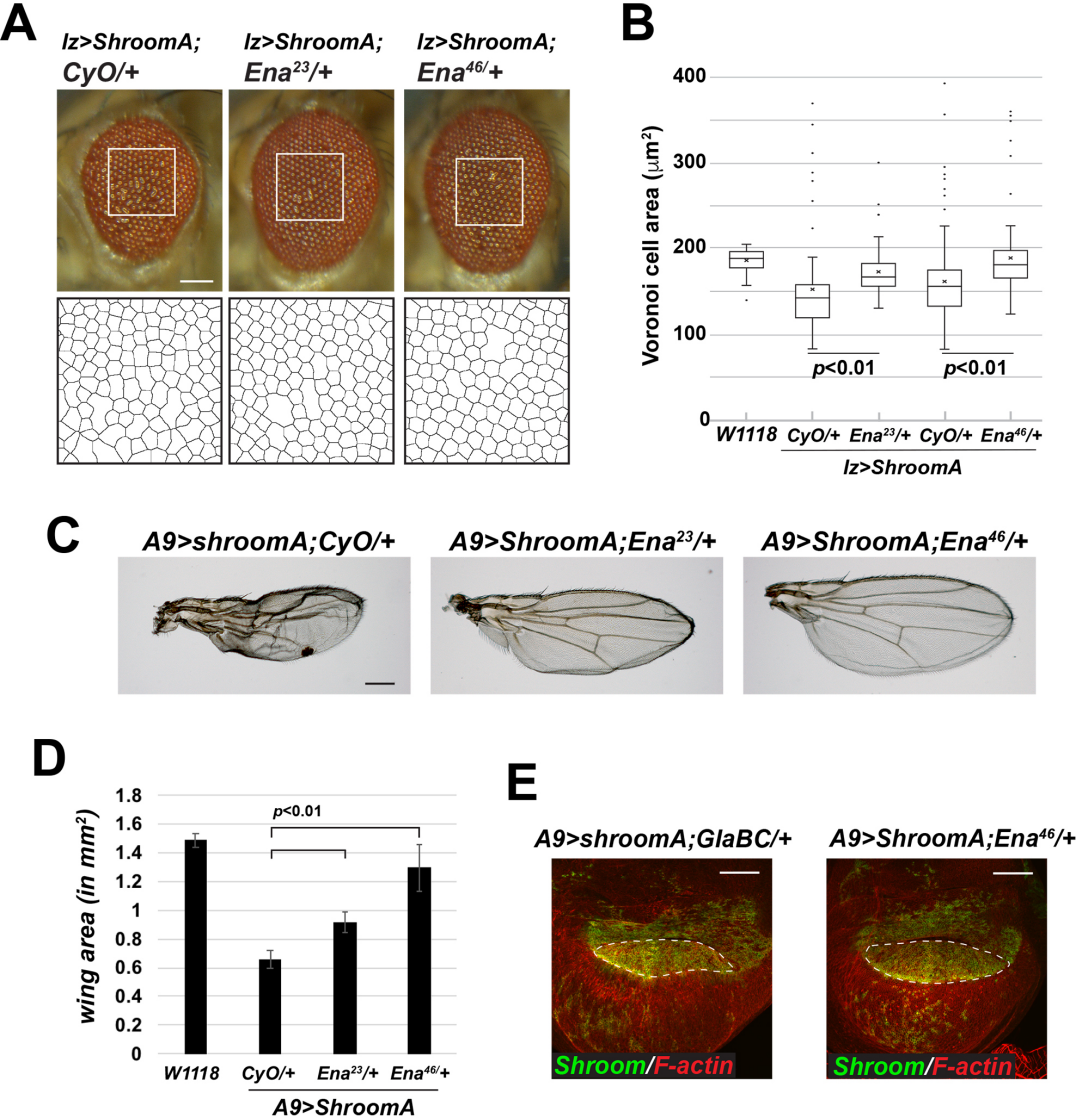
**ShroomA-induced eye and wing phenotypes require Ena.** (A,B) Images of heterozygous F1 male adult eyes of the indicated genotypes raised at 28°C. Boxed regions were used to generate the Voronoi tessellation to quantify the phenotype. (C) Female heterozygous F1 adult wings of the indicated genotypes raised at 25°C. Scale bar: 100 µm. (D) Wing area for the indicated genotypes. *n*=at least 15 wings. Significance was determined using a two-tailed *t*-test, error bars indicate s.d. (E) Imaginal discs from the indicated genetic backgrounds were isolated and stained to detect Shroom and F-actin. Anterior is to the left and dorsal is to the top, dashed line demarcates the dorsal region of the wing pouch, scale bars: 50 µm.

*Enabled* (*Ena*) and *short stop* (*shot*) were identified as suppressors in our initial screen and both regulate cytoskeletal architecture in epithelia. Previous work in *Drosophila* identified Ena as a determinant of actin dynamics that is required for epithelial morphology (Gates et al., 2007). Additionally, it has also been suggested that Ena/VASP are important for Shroom3 induced apical constriction in MDCK cells (Plageman et al., 2010). Shot is a member of the spectraplakin family of actin-microtubule crosslinking proteins and is required for microtubule organization in axons and follicular epithelia in *Drosophila* (Applewhite et al., 2010; Bottenberg et al., 2009; Nashchekin et al., 2016; Takacs et al., 2017). Microtubules have also been implicated in Shroom-regulated cellular architecture (Lee et al., 2007). To further investigate the role of Ena and Shot, we assessed the ability of different alleles to modify the phenotypes in the eyes and wings (Figs 5 and 6). In the eye, we see significant restoration of normal ommatidia organization and distribution (Figs 5A and B, 6A and B), while in the wing we observe a significant increase in area and decrease in the crumpled morphology (Figs 5C and D, 6C and D). As was observed for *Zipper*, the *Shot* and *Ena* alleles exhibit different degrees of suppression of the Shroom phenotypes, relative to each other, although all are predicted to be amorphic or loss-of-function alleles. In the case of *Ena*, both alleles used in this study are nonsense mutations, potentially resulting in truncated proteins, if translated. In the case of *Shot*, the molecular natures of *shot^SF20^* and *shot^3^* are unknown but both are predicted to be strong alleles, while *shot^V104^* encodes a C-terminally truncated protein (Bottenberg et al., 2009; Strumpf and Volk, 1998). These data again point to the importance of verification using multiple alleles.

In an effort to understand the cellular basis for the observed genetic interaction, we stained wing imaginal discs to detect Shroom and Shot. In control discs, Shroom and Shot are both expressed in the anterior wing margin (Fig. 5E). At the subcellular level, while both are apically enriched, they exhibit complementary distribution, with Shot more medial and Shroom more peripheral (arrowheads, Fig. 5E′–E‴). This is consistent with previous descriptions of Shot localization in epithelial cells (Nashchekin et al., 2016). This distribution is largely maintained in cells that express ectopic ShroomA, although there appears to be elevated levels of Shot in cells expressing excess ShroomA (arrows, Fig. 5F–F‴). This indicates that Shroom and Shot are in the same subcellular domain and could cooperate in regulating apical architecture.

To verify that our analysis is not complicated by *CyO*, we balanced the *Ena^46^* and *Zip^2^* alleles over *In(2LR)Gla, wg[Gla-1] PPO1[Bc]* (referred to as GlaBC) and repeated the analysis of the wing phenotype in the F1 progeny (Fig. S2). We observe similar phenotypic suppression in both instances. We utilized this same cross to ensure that suppression is not the result of decreased ShroomA expression. We separated F1 larva based on the presence or absence of the dominant *PPO1[Bc]* marker and stained the wing imaginal discs to detect ShroomA. ShroomA expression in the dorsal wing pouch is comparable in *A9>ShroomA/+;Ena^46^/+* and A9>*ShroomA/+;GlaBC/+* discs. We also observe that the dorsal compartment of the wing pouch from the *A9>ShroomA/+;Ena^46^/+* discs is restored to a more regular morphology (Fig. 6E, dotted lines).

### Colocalization of Ena and ShroomA

**Fig. 7.**
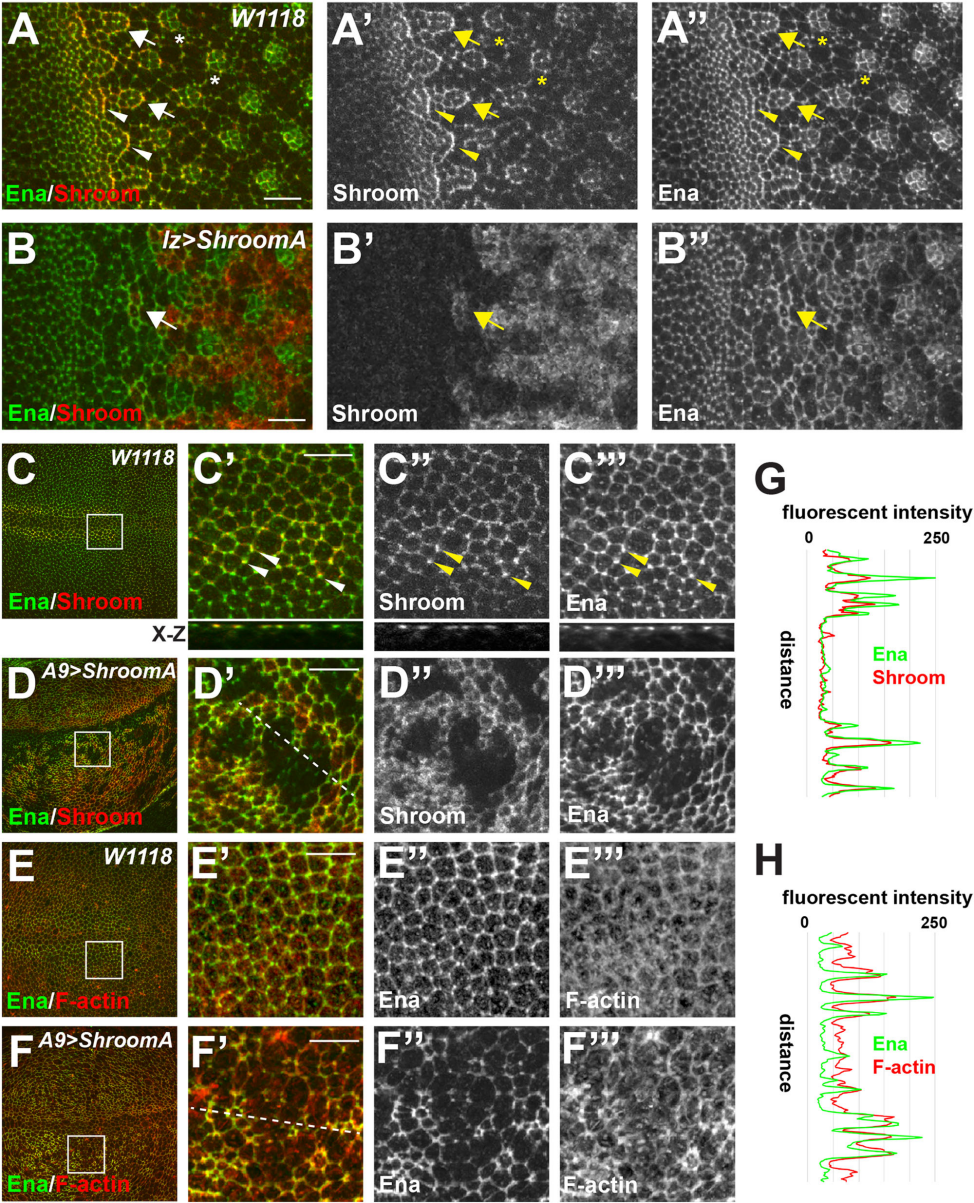
**Colocalization of ShroomA and Ena.** (A,B) Eye imaginal disc from *W1118* (A–A″) or *lz>ShroomA/+* (B–B″) larva stained to detect Shroom and Ena. Anterior is to the left. In A–A″, arrowheads denote colocalization of ShroomA and Ena in cell–cell junctions, arrows indicate colocalization of ShroomA and Ena in the peripheral junctions of ommatidia, asterisk show restriction of ShroomA expression to R3/4. In B–B″, arrows indicate cells overexpressing ShroomA with elevated junctional Ena. (C,D) Wing imaginal discs from *W1118* (C–C‴) or *A9>ShroomA/+* (D–D‴) larva stained to detect Shroom and Ena. X-Z projection is shown beneath C′–C‴. Boxed regions in C and D are shown enlarged in C′–D‴. Arrowheads denote tricellular junctions, anterior to the left and dorsal to the top. Dashed line in D′ was used to generate fluorescent intensity profile. (E,F) Wing imaginal discs from *W1118* (E–E‴) or *A9>ShroomA/+* (F–F‴) larva stained to detect Ena and F-actin. Dashed line in F′ was used to generate fluorescent intensity profile. (G and H) Fluorescent intensity plots of Ena and Shroom (G) or Ena and F-actin (H). Scale bars: 10 µm in all panels.

To further investigate how Ena and ShroomA cooperate to regulate cell and tissue morphology, we stained *W1118*, *lz>ShroomA/+*, and *A9>ShroomA/+* imaginal discs to detect Ena and Shroom. In *W1118* imaginal discs, endogenous Shroom and Ena proteins exhibit extensive overlap in adherens junctions of the lines and arcs that are formed following passage of the morphogenetic furrow (Fig. 7A, arrowheads). Shroom and Ena colocalization is maintained at the periphery of the pre-clusters of cells that will form the ommatidia (Fig. 7A, arrows). As Shroom expression becomes restricted to R3/4, Ena protein is seen in the cell junctions of ommatidia (Fig. 7A, asterisks). In *lz*>*ShroomA/+* eye discs, we observe increased Ena staining in cell–cell junctions of those cells that express ectopic ShroomA but typically express lower levels of Ena and are not incorporated into the ommatidia pre-clusters (Fig. 7B, arrow).

Endogenous Shroom and Ena are widely expressed in the wing disc and both are more highly expressed in the anterior region of the wing margin (Fig. 7C). At the subcellular level, both are localized to apically positioned tri-cellular and cell–cell junctions (Fig. 7C′–C′′′, arrowheads). In A9>*ShroomA/+* imaginal discs, there is a dramatic increase in Ena localization to cell–cell junctions throughout the wing disc. As seen in the eye disc, this increase occurs only in those cells that express ectopic ShroomA (Fig. 7D). To further investigate the role of Ena function in Shroom-induced phenotypes, we stained *W1118* or A9>*ShroomA/+* imaginal discs to detect Ena and F-actin (Fig. 7E,F). Consistent with the known activities of both Ena and Shroom, cells that exhibit increased levels of junctional Ena also display increased levels of junctional F-actin. Analysis of the fluorescent intensity of ShroomA versus Ena and Ena versus F-actin suggests there is a direct correlation between the localization of ShroomA, Ena, and F-actin (Fig. 7G,H). Taken together, these data suggest that ShroomA regulates the distribution of Ena and that ShroomA and/or Ena may function to regulate the amount of junctional F-actin.

### Shroom regulation of Ena localization and the role in apical constriction

**Fig. 8.**
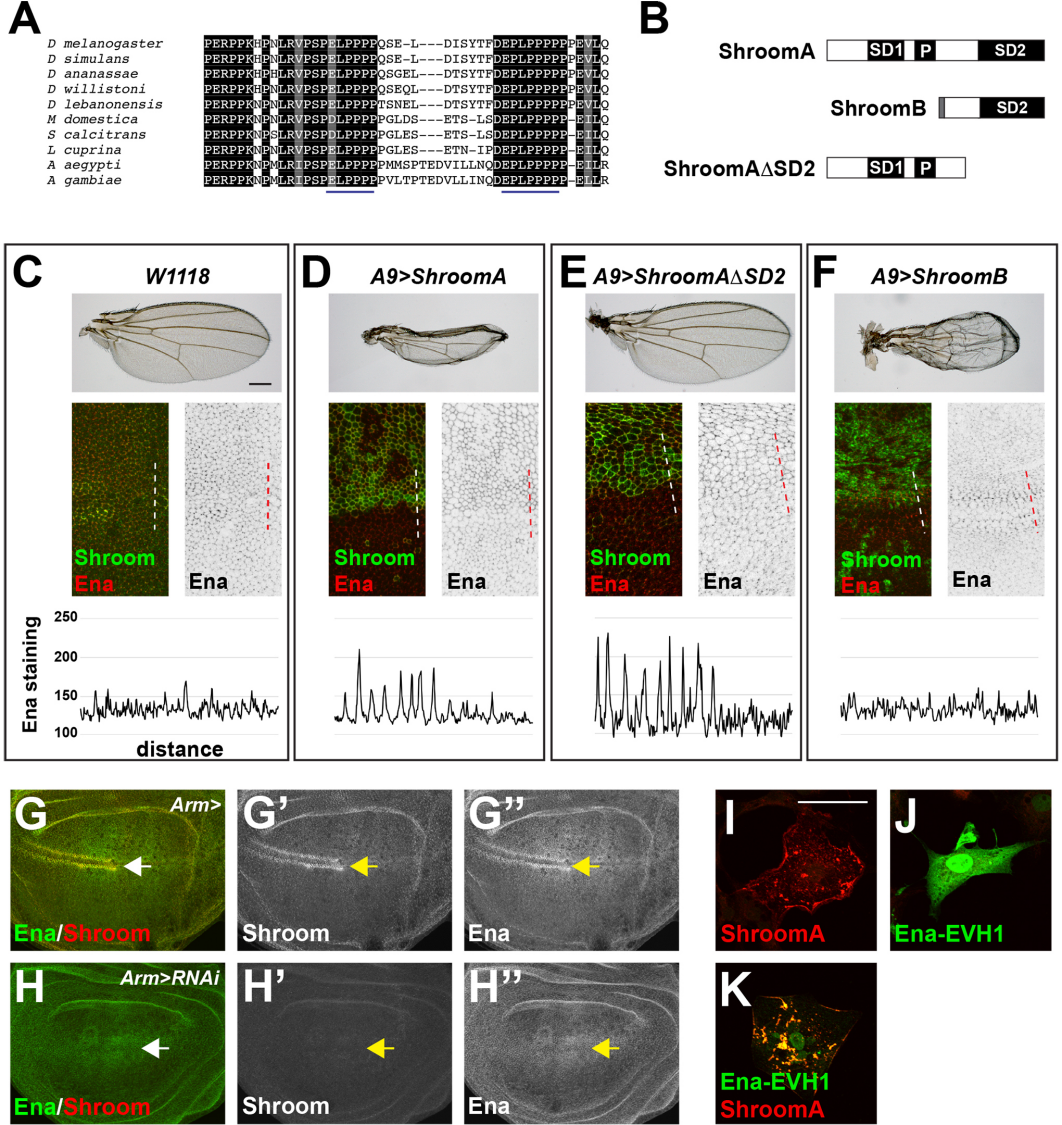
**ShroomA regulates Ena distribution.** (A) Amino acid alignments of ShroomA from multiple insect species shows the conserved proline rich region that contains two putative EVH1 binding sites (underlined). (B) Schematic of ShroomA, ShroomB, and ShroomAΔSD2. s.d., Shroom Domain; P, proline-rich domain. (C–F) Phenotypes and Ena recruitment induced by Shroom isoforms. Top images show the wing phenotypes of adult females of the indicated genotypes raised at 25°C. Middle panels show expression of Shroom isoforms and Ena distribution in wing imaginal discs isolated from the indicated genotypes. Ena staining alone is shown in grey scale. Dashed lines indicate the regions used to determine the fluorescent intensity of Ena staining in Shroom expressing versus non-expressing cells (bottom panels). Scale bar: 50 µm. (G,H) Wing imaginal discs isolated from control *arm>* (G-G″) or *arm>Shroom RNAi* (H–H″) larva and stained to detect Shroom and Ena. Arrow denotes the margin. (I–K) Cos7 cells transiently expressing the Ena-EVH1 domain alone, ShroomA alone, or ShroomA and the EVH1 were stained to detect Shroom and the EVH1. Scale bar: 10 µm.

The above results suggest that Ena activity is necessary for Shroom-induced apical constriction. Additionally, previous studies indicate that the EVH1 domain of *Drosophila* Ena binds to proline-rich ligands of the sequence LPPPP, which is slightly different from the canonical ‘FPPPP’ sequence (Chen et al., 2014). The primary amino acid sequence of ShroomA contains two such motifs, SPELPPPP and DEPLPPPPP, within a stretch of approximately 25 amino acids (Fig. 8A). Based on these observations we wanted to determine if Ena recruitment is dependent on these sequences and if increased recruitment of Ena is sufficient to cause apical constriction. To test this, we expressed variants of Shroom that contain different domains of the protein using the *A9-gal4* driver (Fig. 8B). These include full-length ShroomA, ShroomAΔSD2, a version of ShroomA that retains the actin and putative EVH1 binding sites but lacks the Rok binding motif, and ShroomB, a naturally occurring isoform that retains the SD2 but lacks the actin and putative EVH1 binding sites (Bolinger et al., 2010; Mohan et al., 2012). As expected, *A9>ShroomA* induces a strong wing phenotype and this coincides with increased Ena distribution in the dorsal compartment where ShroomA is expressed (Fig. 8D). Similarly, the ShroomAΔSD2 deletion variant is highly expressed in the dorsal compartment and there is a commensurate increase in Ena localization (Fig. 8E). However, ShroomAΔSD2 is largely inert as the *A9>ShroomAΔSD2* wings exhibit normal morphology. These data suggest that the SD2 is essential for apical constriction while Ena recruitment is needed for ShroomA-induced apical constriction. This is further supported by the observation that ShroomB causes a severe wing phenotype but does not induce significant changes in Ena distribution (Fig. 8F).

To determine if endogenous Ena distribution is regulated by Shroom, we reduced the level of Shroom protein using RNAi. In control wing discs, Shroom and Ena are co-expressed and co-localize in the anterior margin (Fig. 8G, arrow). As expected, the Shroom RNAi effectively reduces the amount of Shroom protein in the wing imaginal disc (Fig. 8H′). Coincident with the loss of Shroom protein, there is a decrease in the amount of Ena protein in the anterior portion of the wing margin (Fig. 8H″, arrow). However, the localization of Ena protein is not perturbed in other regions of the wing disc. Based on the presence of potential EVH1 binding sites in ShroomA, we tested the ability of the EVH1 domain to co-localize with ShroomAΔSD2 in Cos7 cells (Fig. 8I–K). In these cells, ShroomAΔSD2 is localized to the cell cortex and cytoplasmic puncta as previously described (Bolinger et al., 2010) while the EVH1 is largely cytoplasmic. When co-expressed, we observe extensive re-distribution of the EVH1 to ShroomA cytoskeletal compartments (Fig. 8K). Together, these data suggest that ShroomA can regulate the localization of Ena *in vivo* and that this is likely mediated by the EVH1 domain of Ena and the LPPPP sequences of ShroomA.

## DISCUSSION

### *Drosophila* as a model system to identify modifiers of the Shroom pathway

This study describes a genetic approach to identify cellular pathways that participate in tissue morphogenesis. This method takes advantage of the observation that ectopic Shroom protein can utilize the endogenous contractile machinery within epithelial cells to induce apical constriction and disrupt normal tissue morphology. While the work described here focuses on candidate genes that encode known regulators of epithelial and tissue architecture, we predict these tools can be used to perform unbiased, genome-wide screens to identify novel participants in Shroom-mediated cellular processes. We have utilized two different tissues, eye and wing imaginal discs, for these studies and are confident that these screens can identify factors that are used in a wide range of tissues and cells to control cell dynamics. This is based on the observations that ShroomA induces similar cellular phenotypes in both types of imaginal discs and the phenotypes can be modified in both tissues. A powerful aspect of this screen is that these processes are functionally conserved in vertebrate cells and tissues. Additionally, the simplified nature of the *Drosophila* genome makes these screens possible. Due to genetic and functional redundancy, we predict that the analysis performed here would be more complicated using vertebrate or cell culture model systems. *Drosophila* have single genes for *Shroom*, *Rok*, *myosin II*, and *Ena* while mammals possess gene families for these factors. In support of this, we have previously shown that both *Rock1* and *Rock2* must be inhibited to prevent Shroom3-mediated apical constriction in cell culture (Mohan et al., 2013). This screening approach should allow for the identification of novel genetic interactions in *Drosophila* that can be further verified in mammalian model systems to define their potential role in human disease.

### The role of cytoskeletal dynamics in the Shroom pathway

Most of the modifiers we identified participate in defining actin or microtubule architecture. Of these, several regulate actin dynamics at the level of polymerization or stability, including Ena, Diaphanous, Chickadee, and Slingshot. Interestingly, three of these proteins can be linked, directly or indirectly, to neural tube formation in mice (Grego-Bessa et al., 2015; Lanier et al., 1999). It should be noted that several classes of actin regulators did not appear to modify the Shroom phenotypes, including nucleators, binding proteins, or adaptors, suggesting that specific types of actin organization are required for Shroom-induced perturbation of cell architecture. This is further supported by the observation that Tropomyosin was also identified in the screen. Tropomyosin regulates the structure of actin filaments and the binding of other proteins, including myosin II and cofilin, that in turn modulate cell architecture or behavior (Gunning et al., 2015). It is particularly intriguing to note that *Tropomyosin* mutations can suppress phenotypes caused by the loss of *Flapwing*, presumably caused by increased myosin II activity (Vereshchagina et al., 2004). In addition to the actin cytoskeleton, these studies also support a role for microtubules in Shroom-induced phenotypes. This is consistent with the role of microtubules in apical constriction in *Drosophila* (Corrigall et al., 2007; Fernandes et al., 2014; Ko et al., 2019). Recent evidence indicates that apical-medial microtubules play an important role in ventral furrow invagination and this is mediated by Patronin, a protein known to interact with Shot (Ko et al., 2019; Nashchekin et al., 2016). These studies show that microtubules stabilize the connection of contractile networks to cell junctions to facilitate tissue morphogenesis. These studies are consistent with our results in relation to Shroom function and Shot distribution in the wing epithelium. It will be interesting to determine if the identified proteins act upstream or downstream of Shroom. While our data suggest Ena acts downstream of Shroom, proteins such as Tropomyosin could function upstream by regulating the amount of Shroom that can bind to F-actin or downstream by modulating the amount of myosin II that can be recruited or activated by the Shroom-Rok complex. It was surprising that determinants of cell adhesion or polarity, such as cadherins or Par complex proteins, were not identified in this screen. It is possible that these proteins are present in sufficient quantity and reducing the dosage is unable to modify the Shroom overexpression phenotype and thus other genetic approaches will be needed to assess the role of these pathways.

### Shroom expression is highly regulated during tissue morphogenesis

Our data show that endogenous Shroom protein is expressed in epithelial cells during wing and eye development, suggesting it functions in these tissues under normal circumstances. *Shroom* null flies that survive to adults do not exhibit significant defects in the eyes or wings, although null embryos do exhibit defects in convergent extension (Simoes Sde et al., 2014) and perhaps this could contribute to the observed reduction in viability. In embryos, Shroom is important for the polarized distribution of contractile myosin II needed for convergent extension. It is possible that Shroom activity in disc epithelial cells is redundant to other pathways that regulate Rok and myosin II and Shroom normally functions to make these pathways more robust or function with higher fidelity. Uncovering these subtle interactions will require additional genetic approaches. The localization of Shroom in the eye and wing disc appears to be highly regulated and is reminiscent of that exhibited by myosin II and phosphorylated Sqh, particularly in the eye imaginal disc (Escudero et al., 2007; Robertson et al., 2012). We observe a dramatic increase in Shroom protein in cells that are exiting the morphogenetic furrow and forming the pre-clusters that will give rise to the ommatidia. As the ommatidia form, Shroom expression becomes restricted to the R3/4 cells and eventually is lost from these cells. This distribution is essentially the inverse to that of E-cadherin, which is highest in the radial junctions and lower in the circumferential junctions (Fig. 2). This could reflect differences in adhesive interactions between the ommatidia pre-clusters and the inter-ommatidia cells, which facilitates rotation of the ommatidia. This hypothesis is supported by previous studies demonstrating that differential adhesion generates specific cellular organization and compartmentalization in the developing eye (Hayashi and Carthew, 2004; Warner and Longmore, 2009). Interestingly, the PCP protein Flamingo is also expressed in R3 and R4 and we have previously identified interactions between the Shroom3 and PCP pathways in the neural tube (Ho et al., 2010; McGreevy et al., 2015). As eye development continues, we observe Shroom expression in the pigment cells of the pupal retina. In both the imaginal disc and the retina, Shroom distribution is restricted to specific cell junctions, suggesting there are differential adhesive or contractile forces associated with these membranes.

In the wing imaginal disc, we observe expression of Shroom protein in rows of cells that border the anterior half of the wing margin. Consistent with the genetic interactions, we observe a similar expression pattern for both Ena and Shot in these cells. It is currently unclear if the co-expression of Shroom, Ena, and Shot is controlled pre- or post-transcriptionally. It is possible that the expression of *Shroom*, *Ena*, and *Shot* is coordinately regulated in a gene network. Alternatively, the stability or apical localization of these proteins may be interdependent or closely orchestrated. This expression pattern in the anterior wing margin is similar to members of the Irre cell Recognition Module (IRM), including cell surface receptors Roughest, Hibris, and Kirre, which help position the sensory organs (Linneweber et al., 2015). This is particularly interesting in light of the fact that the vertebrate orthologs of these genes, Neph and Nephrin-1, and Shroom3 are all involved in formation of podocytes in the glomerulus of the mammalian kidney (Kestilä et al., 1998; Khalili et al., 2016; Matsuura et al., 2020; Sellin et al., 2003; Yeo et al., 2015). It will be exciting to apply genetic analysis to investigate if these pathways cooperate to regulate tissue morphology.

### The role of Ena in the Shroom pathway

Ena and Shroom show extensive co-expression and colocalization in both the wing and eye imaginal disc, although Ena is more widely expressed than Shroom (Figs 2 and 7). In both the wing and eye imaginal disc, Ena is expressed in most cells and is localized primarily in the tricellular junctions with lower expression in the adherens junctions. However, as seen in the wing margin and the morphogenetic furrow, cells that express Shroom protein also exhibit high levels of Ena in the cell junctions. Importantly, reducing the amount of Shroom protein perturbs the localization of Ena in the anterior wing margin. The relationship between Ena, Shroom, Rok, and myosin II in defining cell shape is likely to be complicated. This stems from the observations that these factors could be placed both upstream and downstream of Shroom. For example, we have previously shown that Shroom distribution to the apical adherens junctions is mediated, at least in part, by direct binding to F-actin. However, it has also been established that RhoA and Rok regulate F-actin architecture to influence Shroom distribution, which then facilitates the polarized distribution of Rok and myosin II (Simoes Sde et al., 2014). Ena has been shown to have multiple roles in *Drosophila* development, including axon guidance, collective cell migration, and epithelial morphogenesis (Gates et al., 2007; Gertler et al., 1995; Jodoin and Martin, 2016; Myat et al., 2019). The role Ena plays in Shroom-mediated apical constriction is unclear. Our data suggest that Ena functions downstream of Shroom and is recruited to adherens junctions via an LPPPP-EVH1 interaction. Ena is primarily defined as a modulator of F-actin dynamics that facilitates the formation of long filaments by competing with barbed-end capping and promoting the addition of actin monomers to the barbed end (Bear and Gertler, 2009). This activity may be important for providing the substrate for activated myosin II to drive cell contraction. This is consistent with studies in vertebrate cells showing that Diaphanous 1, is also required for contractility in adherens junctions (Acharya et al., 2017) and that we also identified *Dia* as a potential modifier of Shroom activity.

### Integration of myosin II into signaling pathways

Elegant studies from several groups have identified many other signaling pathways that control the distribution of contractile myosin II networks during *Drosophila* development, including the Fog, PCP, HH, Dpp, EGF, Toll, and integrin signaling pathways (Corrigall et al., 2007; Dawes-Hoang et al., 2005; Fernandes et al., 2014; Kolesnikov and Beckendorf, 2007; Paré et al., 2019, 2014; Robertson et al., 2012; Winter et al., 2001). How all these signaling pathways are orchestrated and converge on myosin II at the cellular and tissue level is a fascinating question. It has been shown that the above processes use a variety of methods to regulate the small GTPase RhoA, which activates Rok, including several GTP exchange factors or GTPase Activating Proteins (Kolesnikov and Beckendorf, 2007; Mason et al., 2016; Nikolaidou and Barrett, 2004). It should be noted that other GTPases such as Rap1 or CDC42 also regulate apical constriction (Sawyer et al., 2009; Spahn et al., 2012). Our work has shown that Shroom3 may activate Rock independent of RhoA, suggesting that there as mechanisms to bypass small GTPases in the activation of myosin II (Mohan et al., 2013; Zalewski et al., 2016). It will be informative to utilize this screening approach to further test how these pathways might work with ShroomA to control cell morphology.

## MATERIALS AND METHODS

### Fly stocks used and screening approach

Fly stocks were maintained on standard media at 25°C, unless indicated otherwise. Shroom gain-of-function was achieved using the Gal4-UAS system (Brand and Perrimon, 1993). The Gal4 expression drivers, *A9-gal4* and *lozenge (lz)-gal4*, and the *UAS-Shroom* lines have been described previously (Bolinger et al., 2010; Crew et al., 1997; Sun and Artavanis-Tsakonas, 1997). *A9>ShroomA* and *lz>ShroomA* were generated by selecting recombinants between *UAS-ShroomA* and either *A9-gal4* and *lz-gal4*, all of which map to the X-chromosome (Fig. S1), and were maintained as homozygous stocks.

**Table:**
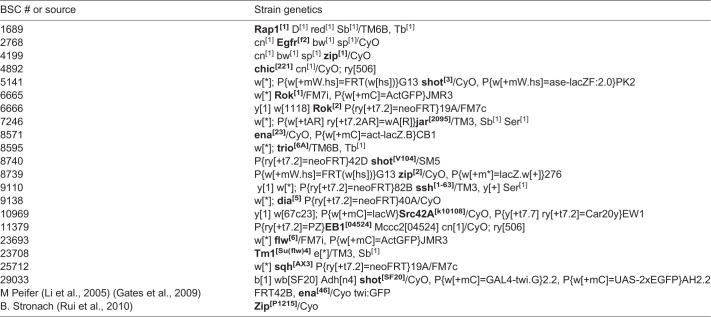

The heterozygous F1 modifier screen was performed as follows. For genes located on the second or third chromosomes, males heterozygous for the candidate alleles were crossed to homozygous *A9>ShroomA* or *lz>ShroomA* females at either 22, 25, or 28°C. The resulting heterozygous F1 progeny were collected and analyzed by measuring the wing blade area or ommatidia organization. For phenotypic analysis, *A9>ShroomA*; *balancer/+* or *lz>ShroomA*; *balancer/+* serve as the controls while *A9>ShroomA; mutant/+* and *lz>ShroomA; mutant/+* are the experimental samples (see Fig. S1 for sample crosses). In all cases, control and experimental categories were also divided based on sex. For candidates on the X-chromosome, females heterozygous for the candidate allele were crossed to *A9>ShroomA* males and F1 progeny collected. *A9>ShroomA/balancer* are the controls and *A9>ShroomA/mutation* are the experimental group. Modification of the wing phenotype was established by calculating the ratio of the areas of the experimental wings to the control wings.

The following alleles were identified as modifiers in these studies:

Other lines used include *In(2LR)Gla, wg[Gla-1] PPO1[Bc]* (GlaBC), y1 w*; *TI{TI} shgGFP* (Bloomington Stock Center), *Shroom* RNAi (VDRC v100672, P{KK106863}), and *Armadillo-Gal4* (a gift from M. Rebeiz, University of Pittsburgh). A complete list of candidate genes and alleles that were used in the preliminary screen can be found in Table S1.

### Immunofluorescence and histology

For wing analysis, adult flies were collected in 70% EtOH and washed through a graded series of EtOH:glycerol to a final solution of 30% glycerol (in PBS). Wings were removed, mounted on microscope slides in 30% glycerol, and imaged with a Leica DMR compound microscope (5X/0.15 air objective) and Leica DFC300F digital camera with Leica Acquire software. Wings were measured and processed using ImageJ and Photoshop. For adult eyes, flies were collected, frozen at −20°C, and imaged using a Leica S8APO microscope with a ring light and polarizing filter. Images were captured using a MC170HD digital camera and Leica Acquire Software and processed using ImageJ and Photoshop. For electron microscopy, adult flies were dehydrated through a grades series of EtOH: hexamethyldisilazane (HMDS) into a final solution of 100% HMDS, sputter coated, and imaged with a Jeol JSM6390LV SEM. To analyze imaginal discs, third instar larva were isolated in PBS/0.1% tween, cut in half, turned inside out, and fixed in 4% paraformaldehyde in PBS/0.1% triton for 20 min. Fixed larva were stained with primary antibodies at 4°C overnight in PBS/0.1% triton, washed in PBS/0.1% triton, and stained with fluorescent secondary antibodies for 2 h at room temperature. Individual imaginal discs were removed from the larva, mounted in Vectashield, and imaged using an Olympus Fluoview confocal microscope with either 40X/1.30 or 100X/1.40 oil immersion objectives. Retinas were dissected 48 h after pupation as described (Hsiao et al., 2012). Antibodies and fluorescent reagents used include Rat anti-Shroom (R11, detects ShroomA and ShroomB, R22, detects ShroomA, 1:250, Bolinger et al., 2010), mouse anti-Myc mAb 9E10, (1:100, a gift from Dr Ora Weisz), mouse anti-Enabled mAb 5G2 (1:200, Developmental Studies Hybridoma Bank), mouse anti-Elav 9F8A9 (1:400, Developmental Studies Hybridoma Bank), mouse anti-Shot mAbRod1 (1:400, Developmental Studies Hybridoma Bank), TRITC-phalloidin (Sigma-Aldrich), and Alexa 488 and 568 goat anti-mouse and goat anti-rat IgG (Invitrogen).

Cos7 cells were maintained at 37°C, 5% CO_2_ in DMEM, supplemented with 10% FBS, pen/strep, and L-glutamine. Cells were transiently transfected with expression vectors using Lipofectamine 2000, plated onto coverslip, and stained to detect Shroom or the myc-tag 24 h post transfection. Sequences encoding the Ena EVH1 domain were amplified by PCR and cloned into pCS3MT. The ShroomA expression vector has been described previously (Bolinger et al., 2010). Cells were imaged using an Olympus Fluoview confocal microscope using a 60X/1.42 oil immersion objective.

## Supplementary Material

Supplementary information
