# Supplementary material for: A modifier screen identifies regulators of cytoskeletal architecture as mediators of Shroom-dependent changes in tissue morphology
Source: Biol Open. 2021 Feb 3;10(2):bio055640. doi: 10.1242/bio.055640 (PMC7875558; doi:10.1242/bio.055640)
Supplement: Supplementary information [file biolopen-10-055640-s1.pdf]

Supplemental Figure 1

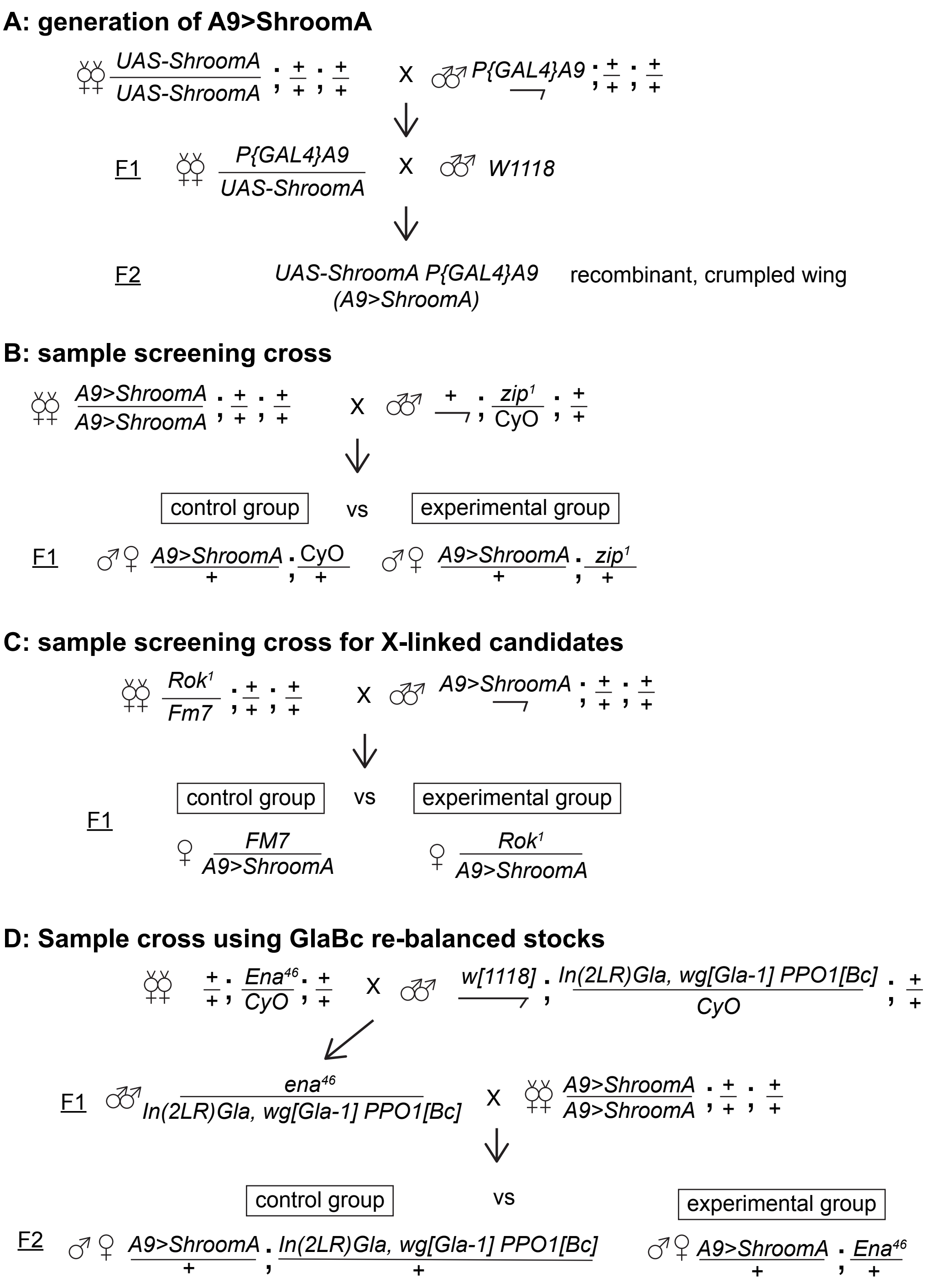

Figure S1. Example genetic crosses used in these studies.
(A) Genetic crosses used to establish *A9>ShroomA* via meiotic recombination. A similar approach was used to generate *lz>ShroomA*.
(B) Example cross outline for F1 heterozygous modifier screen for alleles balanced over *CyO*.
(C) Example cross outline for F1 modifier screen with X-linked candidates.
(D) Example cross using the *In(2LR)Gla, wg[Gla-1] PPO1[Bc]* (GlaBC) balancer.

Supplemental Figure 2

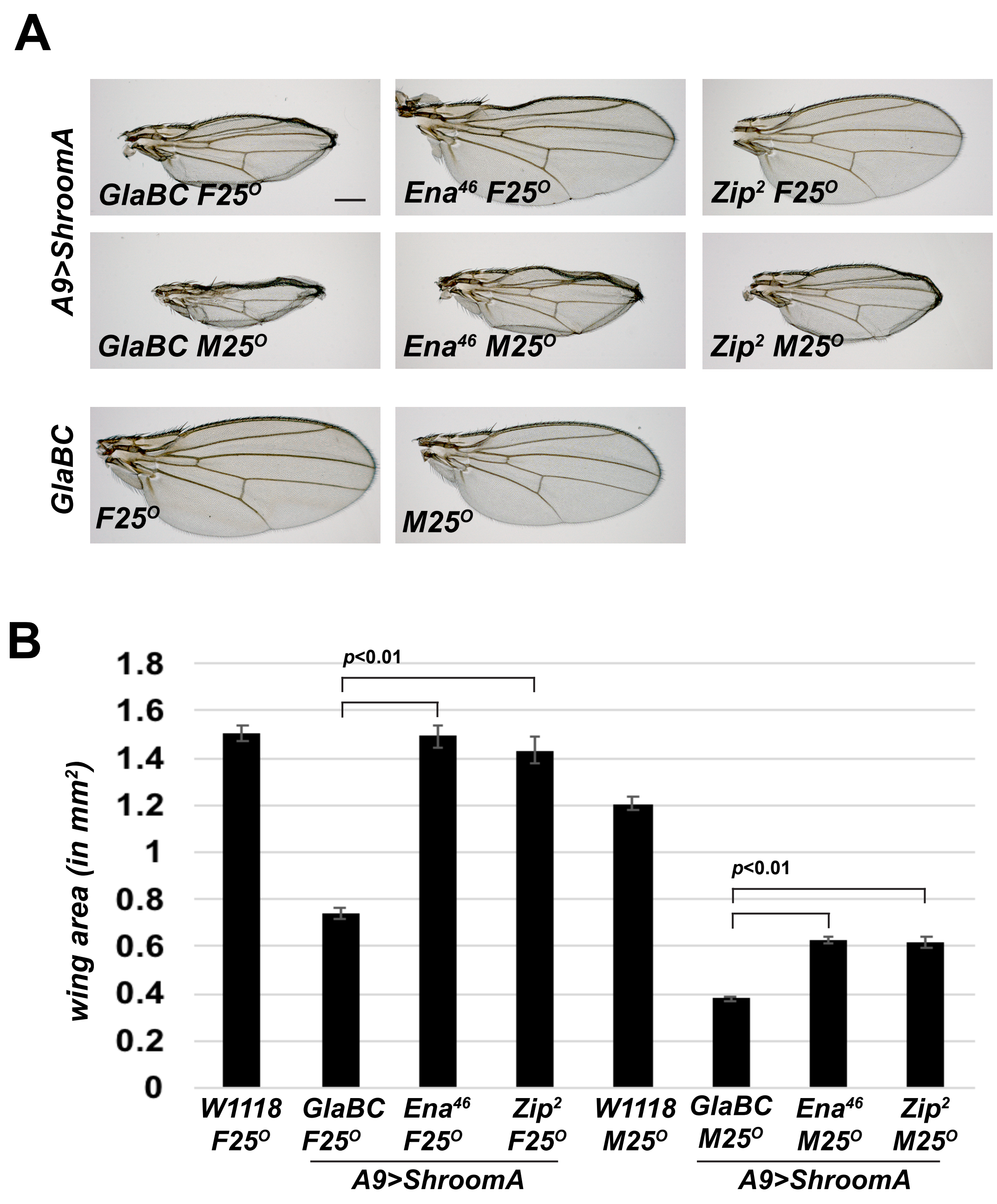

Figure S2. The presence of *CyO* does not influence the genetic analysis of *Ena* and Zip. (A) Crosses were performed with Ena46 and Zip2 balanced over In(2LR)Gla, wg[Gla-1] PPO1[Bc] (GlaBC) at 22°C and 25°C. Images show F1 heterozygous female (F) or male (M) adult wings of the indicated genotypes raised at 22°C or 25°C. Scale bar, 100 μm.
(B) Quantification of wing area shown in (A). Significance was determined using a two-tailed t-test.

Biology Open • Supplementary information

Table S1. List of candidate genes and alleles used in the preliminary screen for modifiers of phenotypes caused by ShroomA expression.

**gene name**	**allele**	**gene name**	**allele**
18 wheeler	18w^{k02701}	*moesin*	moe^{G0323}
abl	abl^{2}	*myospheroid*	mys^{1}
alpha-actinin	Actn^{14}	*pak kinase*	pak^{6}
armadillo	arm^{1}	*par6*	par6^{G796}
arp3	arp3^{EP3640}	*patronin*	patronin^{k07433}
bazooka	baz^{4}	*PDZ-Gef*	PDZ-GEF^{k13720}
btk29	btk29A^{k00206}	*pebble*	pbl^{3}
canoe	cno^{2}	*polychaetoid*	pyd^{1}
cappuccino	capu^{1}	*posh*	posh^{k15815}
capulet	capt^{E593}	*rac1*	rac1^{J11}
cdc42	cdc4^{24}	*raf1*	raf^{7}
chickadee	chic^{221}	*rap1*	rap1^{1}
coracle	cora^{14}	*rho1*	Rho1^{72O}
crumbs	crb^{11A22}	*rhoGEF2*	RhoGEF2$^{4.1}$
dhc64c	Dhc64$^{C4\text{-}19}$	*rok*	rok^{1}
diaphanous	dia^{5}	*rolled*	rl^{1}
disheveled	dsh^{1}	*scar*	scar$^{\Delta 37}$
Drak	Drak$^{[BG00876]}$	*scraps*	scra^{8}
dSor1	Dsor1^{LH110}	*scribble*	scrib^{673}
EB1	EB1^{04524}	*short stop*	shot^{3}
EGFR	egfr^{f2}	*shotgun*	shg^{2}
enabled	ena^{23}	*slingshot*	ssh$^{1\text{-}63}$
expanded	ex^{1}	*spaghetti squash*	sqh^{AX3}
fat	ft^{1}	*spire*	spir^{1}
Fhos	fhos^{01629}	*src42A*	Src42A^{k10108}
flapwing	flw^{6}	*starry night*	stan^{frz3}
flare	flr^{1}	*strabismus*	vang$^{stbm\text{-}6}$
hemipterous	hep^{r75}	*trio*	trio^{6A}
jaguar	jar^{1646}	*tropomyosin*	Tm1$^{Su(flw)4}$
kette	Hem^{03335}	*twinstar*	tsr^{k05633}
lethal giant larval	lgl^{4}	*WASp*	WASp^{1}
Lim kinase	LIMK1^{2}	*yurt*	Yurt$^{87Ek\text{-}7}$
mbs	Mbs^{3}	*zipper*	zip^{1}
merlin	mer^{4}		
